# Trust in AI-Supported Screening in General Practice Among Urban and Rural Citizens: Cross-Sectional Study

**DOI:** 10.2196/69777

**Published:** 2026-02-12

**Authors:** Larisa Wewetzer, Katja Goetz, Soenke Freischmidt, Jost Steinhauser

**Affiliations:** 1 Institute of Family Medicine, University Medical Center Schleswig-Holstein, Ratzeburger Allee 160, Luebeck, 23538, Germany, 49 451 3101 8011

**Keywords:** artificial intelligence, AI, citizens, cross-sectional study, diabetic retinopathy, general practice, trust

## Abstract

**Background:**

The early detection of diseases is one of the tasks of general practice. Artificial intelligence (AI)–based technologies could be useful for identifying diseases at an early stage in general practices. As approximately 90% of the population regularly consults a general practitioner during one year, this could increase the percentage of citizens who take part in meaningful screening measures.

**Objective:**

This study aimed to evaluate the level of trust among citizens in rural and urban areas in AI-supported early detection measures in general practice.

**Methods:**

This cross-sectional study was conducted in the federal state of Schleswig-Holstein, Germany, from November 2023 to December 2023, on the topic of early detection measures with AI in general practice care, among other things. For this purpose, 5000 adult residents of rural areas (Ostholstein, Pinneberg, and Nordfriesland) and urban areas (the city of Kiel) were invited to take part in the survey. Data analysis was carried out using descriptive statistics, subgroup analysis, and linear and stepwise regression analysis to identify the factors that influenced trust in AI-based diagnoses.

**Results:**

Most respondents (787/1790, 44.0%) considered the introduction of an AI-based screening measure to be a sign of modern medicine. Moreover, 21.7% (n=388) of respondents feared that the introduction of such services could lead to a deterioration in the physician-patient relationship. The role of AI in future care was rated as very important by 35.4% (n=634) of respondents. The stepwise regression analysis showed that a positive attitude toward AI in medicine was the strongest predictor (ß=0.420) of trust in AI-based diagnoses. In contrast, trust in physician diagnoses was associated with lower age (ß=–0.111) and shorter waiting times for test results (ß=0.077).

**Conclusions:**

Trust in general practitioner–based diagnoses was approximately 6 times greater than trust in AI applications. Despite concerns about their impact on the physician-patient relationship, approximately one-third of participants believed that the role of AI in health care will grow.

## Introduction

The basic care of all patients in emergency, acute, and long-term care, as well as in prevention and rehabilitation, constitutes a central responsibility of general medicine [1]. Therefore, the early detection of diseases represents an important aspect of secondary prevention in a general practitioner (GP) practice. Appropriate measures can be initiated through early detection, which can considerably improve the course of an individual illness. GPs are often the first to diagnose diseases such as diabetes, hypertension, and cardiovascular diseases [2].

More than 90% of the population regularly consults their GP per year [1], which offers a significant opportunity to increase the proportion of citizens who take part in appropriate screening measures.

Screening for eye diseases is a promising extension of these early detection measures. Diabetic retinopathy (DR), in particular, can lead to severe visual impairment or even blindness if it is not detected and treated early. The number of people in Germany who become blind as a result of DR is approximately 15,000 per year [3]. DR is therefore the second most common cause of blindness [4]. According to a European study, 48% of patients with diabetes do not undergo the recommended screening examination [5].

The current diagnosis of these diseases requires a referral to an ophthalmologist, which can lead to long waiting times, especially in rural areas with limited access to specialists [6].

In this context, artificial intelligence (AI)–supported technologies could play an important role by complementing and extending traditional screening approaches. For example, in general practice, these technologies may include AI-assisted analysis of skin lesions for cancer screening or algorithms that identify abnormal heart rhythms in long-term electrocardiograms. In this study, we focused on DR as an illustrative example.

AI-assisted diagnostic tools could be used to analyze retinal images without requiring the presence of an eye specialist [7,8]. At the same time, recent studies indicate good diagnostic performance of deep learning algorithms. For example, in a study conducted in 2019, the detection of DR was analyzed on 25,326 retinal images of diabetes patients from Thailand. The algorithm showed a sensitivity of 97%, higher than the 74% observed for human experts, and a specificity of 96%, compared with 98% of specialists [9].

The implementation of AI-supported technologies could enable screening for DR directly in the GP practice as part of disease management programs. In this way, suspicious findings could be detected early, and patients could be referred to ophthalmologists accordingly.

It is still unknown whether trust in AI-supported technologies for early disease detection varies across population groups. However, significant variations in attitudes toward eHealth were observed between urban and rural respondents, highlighting a potential disparity in the future implementation of these technologies in general practice across demographic areas [10,11]. Therefore, the aim of the study was to evaluate the level of trust citizens in rural and urban areas have in AI-supported early detection measures in general practice.

## Methods

This cross-sectional study was conducted in accordance with the STROBE (Strengthening the Reporting of Observational Studies in Epidemiology) guidelines [12].

### Study Design and Participants

A cross-sectional study on early detection measures using AI in GP care was conducted in the federal state of Schleswig-Holstein, Germany, from November 2023 to December 2023. To this end, 5000 adult citizens from Schleswig-Holstein were invited to take part in the survey. The citizens were randomly selected from the databases of the local registration office.

Following the first invitation, 2 reminders to participate in the study were sent out at 3-week intervals. A stamped and preaddressed return envelope was included in all mailings to facilitate participation. A short version of the questionnaire was enclosed with the third reminder letter. This approach, which is consistent with the Tailored Design Method in survey research, was chosen to maximize the response rate and reduce the participation barrier for citizens who had not yet responded, by minimizing the time required.

### Data Collection and Questionnaire

Data were collected using a self-constructed questionnaire in paper form. The development of the questionnaire was rigorously based on the findings of a qualitative study that served as a formal pilot phase and informed the generation of items [13]. This preceding qualitative study explored the determinants of AI implementation for DR screening in primary care. In addition, a subset of items—especially those assessing trust in AI vs physician diagnosis and general attitudes toward AI—was pilot-tested in a small qualitative pretest with GPs (1 female GP and 3 male GPs) to ensure clarity and face validity.

To account for different levels of respondent burden, 2 versions of the questionnaire were used:

- The long version consisted of 35 items, covering all conceptual domains in depth (telemedicine use, attitudes toward AI, perceived impact of AI-supported screening, importance ratings for screening attributes, expectations about examination duration, trust in diagnoses, and sociodemographic variables).
- The short version was reduced to 5 core study-related items, capturing only the core constructs (general attitude toward telemedicine, general attitude toward AI in medicine, trust in AI diagnosis, trust in physician diagnosis, age, gender, and region).

The use of long and short questionnaire versions was chosen because shorter instruments in postal surveys can increase response rates and reduce dropout, especially when sent as reminders [14].

The long questionnaire consisted of five sections:

1. Telemedicine experience and attitudes
2. Attitudes toward AI in health care
3. Perceived impact of AI-based screening in general practice
4. Importance of specific aspects of AI-assisted eye disease screening
5. Trust in AI-based diagnosis vs diagnosis by physicians

The participants’ general attitude toward AI applications in general practice was assessed using a 6-point Likert scale ranging from “very positive” to “very negative.” In addition, questions were asked about the expected future role of AI in their own health care and the potential impact of integrating AI into general practice.

Participants were asked to rate from a list of options what impact the use of AI-based screening measures would have on their everyday life and care, such as improving or worsening patient care and physician-patient relationships. In addition, specific aspects were asked about the importance of performing AI-based screening tests. The importance of these aspects was rated on a 6-point scale, ranging from “very important” to “very unimportant.” Finally, respondents were asked about their trust in diagnoses made by AI compared with diagnoses made by GPs.

The questionnaire also included sociodemographic parameters such as age, gender, and regional origin. Sociodemographic information (age, gender, and regional origin) was collected at the end of the instrument. Regional origin was dichotomized into rural (Ostholstein, Pinneberg, and Nordfriesland) and urban (Kiel).

### Statistical Data Analysis

The analyses were carried out using the SPSS software (version 29.0; IBM Corp). The sociodemographic characteristics of the sample (full and short versions of the questionnaire) were analyzed descriptively. Variables with continuous values were summarized using mean values and SDs. Variables with categorical values were represented by absolute (n) and relative (%) frequencies. The regional origin was dichotomized: the regions Ostholstein, Pinneberg, and Nordfriesland were assigned to the rural area, and the Kiel region to the urban area.

The sociodemographic characteristics of the sample (full and short versions of the questionnaire) were analyzed descriptively. Variables with continuous values were summarized using mean values and SDs. Variables with categorical values were represented by absolute (n) and relative (%) frequencies. The regional origin was dichotomized: the regions Ostholstein, Pinneberg, and Nordfriesland were assigned to the rural area, and the Kiel region to the urban area.

The nonparametric Mann-Whitney *U* test was used to compare the full and short versions of the questionnaire regarding the general preferences of telemedicine and AI. Furthermore, a subgroup analysis was performed by using the nonparametric Mann-Whitney *U* test to assess whether the regional origin (0=rural region and 1=urban region) differed concerning the preferences for telemedicine and AI.

Moreover, linear correlations between trust in diagnosis with AI and trust in diagnosis through physicians, the sociodemographic variables, and various influencing factors were calculated using Spearman correlation coefficient. Testing for connections using a correlation is a common procedure to reduce the number of variables for subsequent model calculations. In a further step, significant correlations identified in this way were taken into account in the linear regression models.

Afterward, 2 stepwise regression analyses were performed to evaluate which factors were strongly associated with the outcome variables. Trust in diagnosis with AI and trust in diagnosis through physicians were the outcome variables, while other aspects, such as preferences for telemedicine and AI, attitudes to early detection measures, and some characteristics of participants (such as gender, age, regional origin), were considered potential predictors and handled as covariates. Additionally, the possibility for multicollinearity was considered in both models. The variance inflation factor (VIF) and the tolerance value were reported for the final step of the regression models. Values for VIF should not exceed 5.0, and tolerance values should not be lower than 0.25 [15]. Statistical significance was set at *P*<.05.

### Ethical Considerations

The ethics application for conducting the study was approved by the ethics committee of the University of Lübeck in March 2023 (2023-258_1). All participants provided informed consent prior to participation and were informed that their participation was voluntary and that they could withdraw from the study at any time without consequences. The study involved only the analysis of data collected within the scope of the approved protocol; no additional data were evaluated beyond those covered by the original ethical approval. All data were anonymized prior to analysis, and no personally identifiable information was accessible to the researchers. Appropriate technical and organizational measures were implemented to ensure data protection and confidentiality in accordance with applicable data protection regulations. No incentives were offered for participation.

## Results

### Description of the Sample

Of the 5000 citizens contacted, 1790 (35.8%) took part in the survey, corresponding to a response rate of almost 36%. Of the participants, 785 (43.9%) were male and 972 (54.3%) were female. A total of 10 participants (0.6%) identified themselves as diverse, while 23 (1.3%) did not specify their gender. Of these participants, 352 (19.7%) chose the short version of the questionnaire. Of these, 160 (44.1%) were male, 189 (52.1%) were female, 3 (0.8%) were diverse, and 7 (1.9%) had missing data for this variable. The average age of all participants was 56 years (range 18-96 years).

Of the 1790 participants, 764 (42.7%) lived in the city and 979 (54.7%) lived in a rural area. Most participants lived in Kiel (764/1790, 42.7%), followed by 520 (29.1%) in Ostholstein, 335 (18.7%) in Pinneberg, and 124 (6.9%) in Nordfriesland districts. Table 1 provides further details on the comparison between the long version and the short version of the questionnaire.

**Table 1.** Sociodemographic characteristics of participants (n=1790).

Characteristics^a^		Long version questionnaire (n=1427)	Short version questionnaire (n=363)	*P* value
Gender, n (%)				.64
Female		783 (54.9)	189 (52.1)	
Male		625 (43.8)	160 (44.1)	
Diverse		7 (0.5)	3 (0.8)	
Age (y), mean (SD; range)		55.8 (16.8; 18‐96)	57 (16.4; 18‐91)	.11
Regional origin, n (%)				.09
Kiel (urban)		606 (42.5)	158 (43.5)	
Ostholstein (rural)		406 (28.5)	114 (31.4)	
Pinneberg (rural)		275 (19.3)	60 (16.5)	
Nordfriesland (rural)		109 (7.6)	15 (4.1)	
Assessment of local medical care^b^, mean (SD)		2.97 (1.43)	2.93 (1.32)	.88

a The n values vary due to missing data.

b Ranging from 1 (very good) to 6 (very bad).

### General Attitude, Future Role of AI, and Trust in Diagnosis Through AI and Physicians

A descriptive analysis of general preferences regarding telemedicine and AI in health care was carried out (Table 2). The variables analyzed included general attitudes toward telemedicine and AI, as well as specific aspects such as trust in diagnoses by AI and the future role of AI in one’s own health care.

**Table 2.** General preferences for telemedicine and artificial intelligence (AI)—descriptive analysis.

Variables	Long version, mean (SD)	Short version, mean (SD)	*P* value
General attitude to the topic of telemedicine^a^	3.44 (1.50)	3.58 (1.37)	.06
General attitude to applications of AI in medicine^a^	3.35 (1.49)	3.47 (1.47)	.19
Future role of AI for own health care	2.91 (1.48)	—^b^	—
Trust in diagnosis through AI^c^	3.53 (1.43)	—	—
Trust in diagnosis through physician^c^	1.96 (0.82)	—	—

a Ranging from 1 (very positive) to 6 (very negative).

b Not available.

c Ranging from 1 (very small) to 6 (very big). These items were not asked in the short version.

For the general attitude toward applications of AI in medicine, a mean value of 3.35 (SD 1.49) was observed for the long version of the questionnaire, and a mean value of 3.47 (SD 1.47) was observed for the short version.

The assessment of the future role of AI in personal health care had a mean value of 2.91 (SD 1.48). The SD of 1.48 suggests a wide distribution of responses. It was rated important by 35.4% (n=634) of respondents.

Trust in diagnoses by AI is rated with a mean value of 3.53 (SD 1.43). In comparison, trust in diagnoses by physicians had a mean value of 1.96 (SD 0.82).

### Subgroup Analysis Concerning Regional Origin and General Preferences to Telemedicine and AI

Participants from urban regions were more positive toward telemedicine (mean_urban_ 3.26, SD 1.5; mean_rural_ 3.56, SD 1.5; *P*<.001) and toward applications of AI in medicine (mean_urban_ 3.11, SD 1.4; mean_rural_ 3.52, SD 1.5; *P*<.001) than participants from rural regions. Moreover, for respondents from urban regions, AI was perceived to play a bigger future role for own health care (mean_urban_ 2.65, SD 1.4; mean_rural_ 3.09, SD 1.5; *P*<.001), and they reported greater trust in diagnoses made by AI (mean_urban_ 3.38, SD 1.4; mean_rural_ 3.63, SD 1.5; *P*<.001) than participants from rural regions. No significant difference was observed for trust in diagnosis through physicians (mean_urban_ 1.99, SD 0.80; mean_rural_ 1.93, SD 0.81; *P*=.14).

### Relevance of AI-Based Screening Measures in the GP Practice

**Figure 1.**
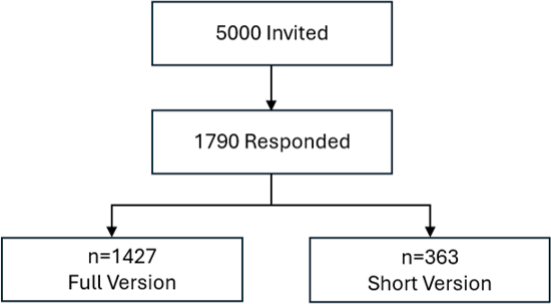
Response rates for the long version vs short version of the questionnaire.

Participants were asked to rate the use of AI-based screening measures in GP practices (Figure 1). Respondents had the opportunity to select several statements that applied.

The analysis of the survey results showed a differentiated perception of the AI-based screening measures, which reflected both potential benefits and concerns among the citizens surveyed.

Out of the 1790 respondents, the most frequently mentioned statement was “signs of modern medicine,” selected by 787 (44.0%) participants. The statement “improved patient care” was selected by 618 participants (34.5%), followed by “easing everyday life” with 445 (24.9%) responses. In contrast, 388 (21.7%) participants stated that they would expect a “worsened doctor-patient relationship,” and 270 (15.1%) respondents described the measure as “intensifying uncertainty.”

The statements “improved doctor-patient relationship” (n=167, 9.3%), “signs of unnecessary technologization” (n=162, 9.1%), “deterioration in patient care” (155, 8.7%), and “difficulty in everyday life” (n=37, 2.1%) were selected less frequently (Multimedia Appendix 1).

### Aspects of the Implementation of Screening Examinations for Eye Diseases Using AI in GP Practices

In the data received from the long version of the questionnaire, the most important factors from the citizens’ perspective were trust in the GP (mean 1.78, SD 1.24) and the reliability of the examination (mean 1.43, SD 1.01), followed by data security (mean 2.19, SD 1.52).

With regard to organizational factors, the accessibility of the practice (mean 2.14, SD 1.31) and the waiting time for an ophthalmologist appointment (mean 2.13, SD 1.26) were also rated as relevant. The protection against additional examination costs (eg, Individual Health Services; mean 2.30, SD 1.42) was rated as moderately important.

Factors considered less important from the citizens’ perspectives included not having their pupils dilated (mean 3.24, SD 1.65), the duration of the examination (mean 2.93, SD 1.45), and the distance to the nearest ophthalmologist (mean 2.50, SD 1.39).

The results showed that trust in the GP conducting the examination, the reliability of the examination, and data security were the most important aspects considered by the participants when implementing such technologies in GP practices (Table 3).

**Table 3.** Evaluation of the aspects of artificial intelligence–supported screening for eye diseases in general practitioner practices.

Variables^a^	Factors, mean (SD; 95% CI)	
Trust in general practitioner	1.78 (1.24; 1.71‐1.86)	
Duration of the examination	2.93 (1.45; 2.84‐3.02)	
Waiting time until the test results are available	2.44 (1.29; 2.37‐2.52)	
Data security	2.19 (1.52; 2.10‐2.29)	
Reliability of the examination	1.43 (1.01; 1.37‐1.49)	
Not having the pupils dilated	3.24 (1.65; 3.14‐3.35)	
Accessibility of the practice	2.14 (1.31; 2.06‐2.22)	
Distance to the nearest ophthalmologist	2.50 (1.39; 2.41‐2.58)	
Waiting time for an eye specialist appointment	2.13 (1.26; 2.05‐2.21)	
Waiting time in an ophthalmologist’s practice	2.49 (1.31; 2.41‐2.57)	
Protection against additional examination costs	2.30 (1.42; 2.22‐2.39)	
Performance of the examination by medical assistants	2.12 (1.30; 2.04‐2.20)	

a Scale from 1=very important to 6=very unimportant.

### Factors Associated With Trust in Diagnosis With AI

The stepwise regression analysis is presented in Table 4 and reports only coefficients with statistical significance at the *P*<.05 level. A 9-step model was fitted and explained more than 60% (*R*²=0.602) of the variance in the dependent variable, “trust in diagnosis through AI.” In the first step of the stepwise regression analysis, the variable “general attitude to applications of AI in medicine” showed the highest explained variance (*R*²=0.509). A higher positive level of attitude toward applications of AI in medicine was strongly associated with higher level of trust in diagnosis through AI. Individual characteristics were not included in the regression model, as *P*>.05. Collinearity statistics ranged between 2.112 (VIF value) and 0.473 (tolerance value) for “general attitude to applications of AI in medicine” and 1.140 (VIF value) and 0.877 (tolerance value) for “improved patient-physician relationship.”

**Table 4.** Associations of individual characteristics, general preferences for telemedicine and artificial intelligence (AI), and attitudes toward early detection measures in general practitioner care with general trust in AI (results of stepwise linear regression analysis).

Variables^a^	β	*P* value
General attitude to applications of AI in medicine	0.420	<.001
Intensifying uncertainty	0.136	<.001
Future role of AI for own health care	0.164	<.001
Signs of unnecessary technologization	0.120	<.001
Improved physician-patient relationship	−0.063	.003
Providing security	−0.091	<.001
Duration of the examination	0.063	.004
Worsened physician-patient relationship	0.066	.004
Reliability of the examination	0.053	.02

a Last step was reported.

### Factors Associated With Trust in Diagnosis Through Physicians

The stepwise regression analysis is presented in Table 5 and reports only coefficients with statistically significances at the *P*<.05 level. A 5-step model was fitted and explained more than 4.8% (*R*²≈.048) of the variance in the dependent variable, “trust in diagnosis through physicians.” For example, a higher positive level of trust in diagnosis through physicians was strongly associated with lower age and greater importance attributed to the waiting time until the test results were available. Collinearity statistics ranged between 1.308 (VIF value) and 0.765 (tolerance value) for “distance to the nearest ophthalmologist” and 1.007 (VIF value) and 0.993 (tolerance value) for “providing security.”

**Table 5.** Associations of individual characteristics, general preferences for telemedicine and artificial intelligence, and attitudes toward early detection measures in general practice care on general trust in diagnosis through physicians (results of stepwise linear regression analysis).

Variables[Table-fn T5_FN1]	β	*P* value
Age of the participants	−0.111	<.001
Waiting time until the test results are available	0.077	.03
Providing security	0.093	.003
Local general medical care	0.089	.005
Distance to the nearest ophthalmologist	0.090	.01

a Last step was reported.

## Discussion

This study examines citizens’ attitudes toward the use of AI in general practice, particularly in the context of early detection measures. The study showed that the introduction of AI-supported technologies is met with mixed reactions.

### Differences Between Rural and Urban Areas

Significant differences emerged between urban and rural participants. People from urban areas were significantly more positive about the use of AI in medicine than those from rural areas.

Differences in access to medical services between urban and rural areas are deeply rooted and reflect a complex mix of infrastructural, socioeconomic, and cultural factors [16,17]. While urban areas often provide a wide range of medical services and specialists, some rural areas experience a shortage of physicians, greater distances to health facilities, and limited access to specialized treatment [17].

The introduction of AI and telemedicine is often promoted as a solution to these inequalities by extending access to care. However, emerging research suggests that these technologies are not always met with acceptance [10,11]. The reasons for this are varied and include technological barriers, a lack of trust in digital diagnostic methods, and a preference for in-person physician-patient interactions [12,18,19].

To ensure equal access to care, a deeper understanding of the specific needs and concerns of the rural population is needed. This means not only providing technology but also taking into account the cultural and psychological aspects that influence attitudes toward technology.

### Dynamics of Trust in General Practice Care and Technology

This study pointed to a trust deficit in AI-supported technologies. Trust plays an essential role both in interpersonal interactions and in the use of technology. The patient-physician relationship is built on trust, particularly in the physician’s commitment to voluntary responsibility and compassionate judgment [20]. This dimension of trust is crucial, as patients in vulnerable health situations depend heavily on the expertise and care of their treating physicians.

This trust is primarily shaped by the quality of the physician-patient relationship, in particular effective communication, interpersonal care, and the physician’s knowledge of the patient’s individual needs. The study emphasized that these factors play an important role in strengthening patient trust, while other aspects, such as the duration of registration at a practice or the frequency of visits, had less influence on trust [21].

Interpersonal interaction and trust in the treating physician are essential for patient satisfaction and adherence to treatment [20]. Research also highlighted that perceived empathy and individualized care are particularly important to patients, reinforcing the importance of these qualities in maintaining trust in medical professionals [21].

Central to this trust is the concept of continuity of care, particularly relational continuity, in which patients consistently see the same physician over time.

The association between continuity of primary care and patient mortality has been described by categorizing continuity into 3 types: relational continuity, informational continuity, and management continuity. A review of 13 quantitative studies found that greater continuity of care is significantly associated with lower all-cause mortality, particularly among older patients and those with chronic conditions. This protective effect was explained by improved physician knowledge, increased patient confidence, and better adherence to medical advice [22].

Therefore, in an increasingly digitalized world, it is essential that new digital health applications are designed from a patient-centered perspective.

Trust in technologies is predominantly based on functional aspects, such as reliability, security, and predictability [23]. From the perspective of the citizens in our study, the reliability of the examination was the most important factor.

Trust in technology is a construct that is strengthened by a combination of experiences with the technology, trust in users, and trust in regulators. Each of these components plays a critical role in maintaining and fostering trust over time. Past experiences, especially the reliability of the technology, had a significant impact on user trust [24]. In addition, trust is strengthened by the integrity and competence of those who use the technology, especially in the medical field [25], as well as by trust in regulatory authorities responsible for the safety and effectiveness of the technology [26].

The ways in which AI technologies work are currently incomprehensible to the public. This technological opacity requires a certain degree of explainability and interpretability [27]. This lack of transparency means that damage caused by interaction with an AI system cannot be easily proven or clearly attributed [28]. This poses a major challenge and underlines the urgency of developing mechanisms that clearly regulate responsibility and liability in connection with AI decisions.

AI is often seen as a potential solution to current challenges in the health care system, such as relieving the burden on physicians and promoting a person-centered physician-patient relationship [29,30]. In a systematic analysis of 45 studies, the impact of AI on the person-centered physician-patient relationship was investigated. The researchers identified various ways in which the use of AI tools could promote person-centered care. One of these ways was the use of AI tools in a supportive role [31].

The rising interest in AI for medical applications should not overshadow the need for proper scientific review and evaluation. It is crucial that the medical field maintains its high standards of evidence and ethical responsibility to ensure both the credibility and effectiveness of patient care in an increasingly digital world.

While a large body of research demonstrates the benefits of primary care, there is comparatively less evidence for the use of AI technologies in health care. This discrepancy has substantial implications for evidence-based medicine, where clinical decisions should be based on solid scientific research [32].

A lack of uniform evaluation protocols introduces several challenges, such as doubts concerning the long-term effectiveness, safety of patients, security of data, and ethical dilemmas. To integrate telemedicine and AI into health care systems responsibly and effectively, we need flexible and comprehensive evaluation methods that conform to clinical objectives and regulatory guidelines [33].

The literature highlights that the factors influencing health are complex and influenced by a variety of factors [34,35]. Future studies should continue to explore how aspects beyond technology influence health to ensure that the design of new health systems considers a balanced integration of digital and human resources.

### Future Role of AI in Medical Care

While concerns remain about the integration of AI in health care, a significant proportion of participants believed that AI-based early detection measures in general practice could enhance patient care. The application of patient-specific medical data combined with AI assistance systems has the potential to improve treatment outcomes by facilitating prevention, early diagnosis, and individualized therapies. Such advancements could lead to the identification of new medical correlations and innovative approaches to prevention [36].

However, despite these potential benefits, significant challenges and concerns remain. The use of AI necessitates access to substantial amounts of sensitive medical data, raising concerns about data privacy and security. Robust security protocols are essential to safeguard patient privacy and prevent data misuse [37].

The development and integration of AI in health care also bring significant ethical considerations, particularly around accountability for poor decisions or unexpected outcomes. Establishing clear guidelines and defining responsibilities are essential to address these ethical concerns and build trust with patients [38].

In this context, the European Union’s “AI Act” represents a critical step toward creating uniform regulations for AI use within the European Union to ensure safety and trustworthiness. This regulation uses a risk-based approach, categorizing AI applications into different risk levels, with high-risk applications, such as those often found in the medical field, subjected to strict requirements. These requirements include transparency, security, and the avoidance of discrimination, all aimed at ensuring that AI systems in the health care sector adhere to high-quality standards and guarantee patient safety [39].

### Strengths and Limitations

With a response rate of approximately 36%, the study provides a robust insight into the attitudes and opinions of the population of Schleswig-Holstein on the use of AI in GP practices.

The study was limited to Schleswig-Holstein, which could limit the generalizability of the results to the whole of Germany. Furthermore, the sample could be biased by self-selection of participants who may have had a greater interest in or familiarity with digital technologies. Future studies should therefore aim for a broader geographical spread to improve the representativeness and applicability of the results to the entire German population.

### Conclusions

These findings suggest that despite the growing importance of AI in some areas of health care, the traditional physician-patient relationship is seen as absolutely essential. This emphasizes the enduring value of the human elements in health care—personal interaction, empathy, and understanding—features that AI cannot replicate.

Nevertheless, the introduction of AI is still recognized as a sign of modern medicine. People in urban areas are somewhat more open to AI technologies in health care. The integration of AI into health care raises crucial considerations regarding the principles of evidence-based medicine. Balancing technological innovation with the fundamentals of patient-centered care will be crucial in navigating the future landscape of health care.

## Supplementary material

10.2196/69777 Multimedia Appendix 1 Evaluation of an artificial intelligence–based screening measure in general practice.

## Abbreviations

AI: artificial intelligence
DR: diabetic retinopathy
GP: general practitioner
STROBE: Strengthening the Reporting of Observational Studies in Epidemiology
VIF: variance inflation factor
