# Supplementary material for: Trust in AI-Supported Screening in General Practice Among Urban and Rural Citizens: Cross-Sectional Study
Source: JMIR Med Inform. 2026 Feb 12;14:e69777. doi: 10.2196/69777 (PMC12900275; doi:10.2196/69777)
Supplement: Multimedia Appendix 1 [file medinform-v14-e69777-s001.docx]

|  | Frequency (n) | |
| --- | --- | --- |
| Difficultiy in everyday life | 37 |  |
| Deterioration in patient care | 155 |  |
| Signs of unnecessary technologization | 162 |  |
| Improved doctor-patient relationship | 167 |  |
| Fueling uncertainty | 270 |  |
| Providing security | 331 |  |
| Worsened doctor-patient relationship | 388 |  |
| Easing everyday life | 445 |  |
| Improved patient care | 618 |  |
| Signs of modern medicine | 787 |  |

Table 1: Evaluation of an AIartificial intelligence-–based screening measure in general practice
